# Bone regeneration at extraction sockets filled with leukocyte-platelet-rich fibrin: An experimental pre-clinical study

**DOI:** 10.4317/medoral.25462

**Published:** 2022-08-17

**Authors:** Gene Park, Ernesto B Benalcázar Jalkh, Daniel Boczar, Edmara TP Bergamo, Heoijin Kim, Gregory Kurgansky, Andrea Torroni, Luiz F Gil, Estevam A Bonfante, Paulo G Coelho, Lukasz Witek

**Affiliations:** 1 DDS. Department of Biomaterials, New York University College of Dentistry, New York, NY USA; Division of Periodontology, The Ohio State University College of Dentistry, Columbus, OH USA; 2 DDS, MSci, PhD. Department of Prosthodontics and Periodontology, University of Sao Paulo, Bauru School of Dentistry, Bauru, Brazil; Department of Biomaterials, New York University College of Dentistry, New York, NY USA; 3 MD. Hansjörg Wyss Department of Plastic Surgery, NYU Langone Medical Center, New York, NY USA; 4 DDS, PhD Department of Prosthodontics and Periodontology, University of Sao Paulo, Bauru School of Dentistry, Bauru, Brazil; Department of Biomaterials, New York University College of Dentistry, New York, NY USA; 5 DDS. Department of Biomaterials, New York University College of Dentistry, New York, NY USA; 6 BSci. Department of Biomaterials, New York University College of Dentistry, New York, NY USA; NYIT College of Osteopathic Medicine Old Westbury, NY USA; 7 MD, PhD. Hansjörg Wyss Department of Plastic Surgery, NYU Langone Medical Center, New York, NY USA; 8 DDS, MS, PhD. Department of Morphological Sciences, Federal University of Santa Catarina, Brazil; 9 DDS, MSci, PhD Department of Prosthodontics and Periodontology, University of Sao Paulo, Bauru School of Dentistry, Bauru, Brazil; 10 MD, DDS, PhD, MBA. University of Miami Miller School of Medicine, Miami, FL, USA; Department of Biomaterials, New York University College of Dentistry, New York, NY USA; Department of Mechanical and Aerospace Engineering, New York University Tandon School of Engineering, Brooklyn, NY; Hansjörg Wyss Department of Plastic Surgery, NYU Langone Medical Center, New York, NY USA; 11 MSci, PhD. Department of Biomaterials, New York University College of Dentistry, New York, NY USA; Department of Biomedical Engineering, New York University Tandon School of Engineering, Brooklyn, NY USA

## Abstract

**Background:**

We aimed to histomorphometrically evaluate the effects of Leucocyte-Platelet-Rich Fibrin (L-PRF), with and without the combination of a bone grafting material, for alveolar ridge preservation using an *in vivo* canine model.

**Material and Methods:**

Seven dogs (Female Beagles, ~18-month-old) were acquired for the study. L-PRF was prepared from each individual animal by drawing venous blood and spinning them through a centrifuge at 408 RCF-clot (IntrasSpin, Intra-Lock, Boca Raton, FL). L-PRF membranes were obtained from XPression fabrication kit (Biohorizons Implant Systems, Inc., AL, USA). A split mouth approach was adopted with the first molar mesial and distal socket defects treated in an interpolated fashion of the following study groups: 1) Empty socket (negative control); 2) OSS filled defect 3) L-PRF membrane; and 4) Mix of Bio-Oss® with L-PRF. After six weeks, samples were harvested, histologically processed, and evaluated for bone area fraction occupancy (BAFO), vertical/horizontal ridge dimensions (VRD and HRD, respectively), and area of coronal soft tissue infiltration.

**Results:**

BAFO was statistically lower for the control group in comparison to all treatment groups. Defects treated with Bio-Oss® were not statistically different then defects treated solely with L-PRF. Collapsed across all groups, L-PRF exhibited higher degrees of BAFO than groups without L-PRF. Defects filled with Bio-Oss® and Bio-Oss® with L-PRF demonstrated greater maintenance of VRD relative to the control group. Collapsed across all groups, Bio-Oss® maintained the VRD and resulted in less area of coronal soft tissue infiltration compared to the empty defect. Soft tissue infiltration observed at the coronal area was not statistically different among defects filled with L-PRF, Bio-Oss®, and Bio-Oss® with L-PRF.

**Conclusions:**

Inclusion of L-PRF to particulate xenograft did not promote additional bone heading at 6 weeks *in vivo*. However, we noted that L-PRF alone promoted alveolar socket regeneration to levels comparable to particulate xenografts, suggesting its potential utilization for socket preservation.

** Key words:**L-PRF, bone healing, socket preservation.

## Introduction

The medical community has investigated treatment modalities to rehabilitate functional dentition lost due to pathologies or trauma. The removal of a tooth from the alveolar bone leads to a fast bone remodeling process that may compromise the restorability of the edentulous site and jeopardize prosthodontic therapy (1). Alveolar ridge preservation is a technique intended to minimize alveolar bone loss following tooth extraction in an effort to yield a predicTable tridimensional (3D) placement of dental implants while reducing the need for additional bone grafting procedures (1). Therefore, immediately grafting extraction sites may facilitate bone regeneration potentially improving the esthetic outcome of the final restoration (2).

Different types of bone grafts for ridge preservation have been proposed, including autograft, allograft, xenograft, or alloplastic materials (3). Autografts remain the gold standard for bone grafting procedures, but they have disadvantages such as the limited sources, need of a secondary surgical site, and increased risk of postoperative complications (4). However, xenografts are options with abundant availability and slow resorption rate that could maximize maintenance of tissue volume during socket preservation (5). Deproteinized bovine bone has been commonly utilized to induce physiological bone remodeling and increase bone regeneration due to its osteoconductive capacity and chemical and physical similarities to human bone (6).

Modern bone grafting materials and techniques have led to favorable bone regeneration outcomes, but its potential has been limited due to long post-operative healing times, particularly in populations with medical co-morbidities that diminish wound healing capacity (7).

Blood-derivate concentrates have been studied in bone regeneration due to their potential to promote wound healing (8). In the 1970s, Matras *et al*. described the use of fibrin glue to improve skin wound healing in a rat model (9). Later on, upgraded versions such as “platelet-fibrinogen-thrombin mix’ and ‘gelatin platelet-gel foam”, offering higher concentrations of platelets, were introduced (8). Platelet-rich plasma (PRP) was introduced by Marx and Whitman in the late 1900’s. Its high platelets concentration, platelet-derived growth factor (PDGF), transforming growth factor (TGF-B), and vascular endothelial growth factor (VEGF) makes it a viable material for enhancing bone regeneration as an adjunct to bone grafts and socket preservation therapy (10). However, PRP clinical usage has been limited by its arduous handling, fast polymerization, and the possibility of bovine thrombin host reaction (11).

Leucocyte-Platelet-Rich Fibrin (L-PRF) is a second-generation platelet concentrate described by Choukroun and Dohan in 2000. It is produced without biochemical blood manipulation and contains three primary components: 1) platelets and their activated growth factors embedded into a fibrin matrix during a natural polymerization process, 2) leucocytes and cytokines, and 3) dense and complex fibrin matrix without the addition of anticoagulant or gelling agents (12). L-PRF is prepared by drawing blood into a tube without anticoagulant or thrombin, that is immediately subjected to centrifugation. While nearly identical growth factors are contained in PRP and L-PRF, the latter has been associated with superior bone regeneration and maturation, and epithelialization in post-extraction sockets. Furthermore, L-PRF has a simpler harvesting, easier handling, absence of bovine thrombin, slow polymerization, and higher concentrations of leukocytes relative to PRP. As a result, L-PRF preparation has become the primary technique to acquire large amounts of structurally durable blood derived concentrates (13).

Although it is well known that platelet - granules contain a plethora of growth factors such as PDGFs, TGF-, VEGF, and epidermal growth factor (EGF), the biological properties of concentrates and their release kinetics have not been well understood/established (14). To date, only one FDA-approved L-PRF system exists, the Intra-Spin L-PRF centrifuge system in combination with the Xpression preparation box (BioHorizons®, Birmingham, AL, USA). Still, methodology of L-PRF preparation is inconsistent among studies, including variable centrifugation protocols, which may notably impact physical and biological aspects of the L-PRF concentrate, including its volume, cell count, growth factors, and fibrin architecture.

L-PRF concentrates have been clinically utilized for diverse regenerative procedures, such as periodontal surgery, treatment of furcation defects, alveolar preservation following tooth extraction, sinus floor augmentation, soft and hard tissues regeneration, and repair of chronic rotator cuff tears (15). However, there is limited scientific evidence to fully support its effectiveness. A PUBMED literature search of “L-PRF” and “Ridge Preservation” demonstrated that clinical studies vastly outnumber pre-clinical trials conducted since the technique was popularized in the 2000s. This lack of systemic investigation on the use of L-PRF as it relates to bone grafting does not permit for evaluation of bone regeneration, formation, and quality due to the inherent limits of clinical studies. Hence, the paucity of baseline knowledge on L-PRF has led to research that was primarily conducted in a single variable fashion.

Despite being desirable to promote and hasten bone regeneration, the relative contribution of L-PRF when used with grafts or alone have rarely been explored, causing uncertainty about the possible benefit of its use. Therefore, the aim of this study was to histomorphometrically evaluate the effects of L-PRF alone and in combination with a bone grafting material at socket-filled extraction sites in a canine model after 6 weeks *in vivo*. The postulated hypothesis was that the addition of L-PRF to grafting material would yield a higher degree of bone regeneration.

## Material and Methods

Following ARRIVE guidelines and approval of the bioethics committee for animal experimentation (Ecole Nationale Veterinaire D’Alfort, Paris, France). Seven dogs (Female Beagles, ~18-month-old), were acquired for the study. After a one-week acclimation period, surgery was performed under general anesthesia. The preanesthetic medication consisted of an intramuscular (IM) administration of acepromazine maleate (0.2mg/kg), diazepam (0.5mg/kg), and fentanyl (4mg/kg). Anesthetic induction was achieved through ketamine (3mg/kg) while general anesthesia was attained and maintained by 1-2% halothane.

The surgical procedure started with bilateral extraction of the first mandibular molars. Mucoperiosteal flaps were elevated and teeth were sectioned buccolingually for a non-traumatic individual root extraction using root elevators and forceps. We adopted a split mouth approach with the mesial and distal socket defects treated in an interpolated fashion under one of the following study groups prior to standard soft tissue suture closure: 1) Empty socket (spontaneous healing, as a negative control); 2) Bio-Oss® filled defect (Bio-Oss® grafting material); 3) L-PRF filled defect (L-PRF membrane); and 4) OSS w/L-PRF filled defect (combination of Bio-Oss® and L-PRF membrane) (16).

L-PRF was prepared from each individual animal by drawing venous blood and spinning them through a centrifuge at 408 RCF-clot (IntrasSpin®, Intra-Lock, Boca Raton, FL). Subsequently, L-PRF membranes were obtained from XPression® fabrication kit (Biohorizons Implant Systems®, Inc., AL, USA). Postsurgical medication included IM administration of antibiotics (Cefazolin 30mg/kg every twelve hours for three days) and anti-inflammatory (0.2mg/kg per day for three days). Euthanasia was conducted by anesthesia overdose six weeks after socket preservation.

- Histological Preparation and Histomorphometry

At the time of euthanasia, the mandibles were retrieved by sharp dissection and removed en-bloc. The mandibles were stored in 10% buffered formalin solution for 24 hours and dehydrated in a series of 70-100% ethanol solution for several days. After dehydration, the samples were embedded in a methacrylate-based resin (Technovit 9100, Kulzer & Co, Wehrheim, Germany) following to the manufacturer’s protocol. Subsequent to complete polymerization under UV light, the sample blocks were sectioned in a buccal-lingual direction by diamond blade sectioning (Buehler Isomet Low Speed Saw/Isocut Diamond Wafering Blade, Lake Bluff, IL, USA), glued onto glass slides, and polished by SiC abrasive papers (400,600, 800, 1200, and 2400 Grit) in a grinding/polishing machine (Metaserv 3000, Buehler, Lake Bluff, IL, USA) under water irrigation to a final thickness of ~100uM. The samples were then submersed in Stevenel’s Blue and Van Gieson’s picrofuchsin stain, respectively. The percentage of bone area fraction occupancy (BAFO), vertical/horizontal ridge dimensions, and area of coronal soft tissue infiltration was acquired at 50x magnification (Leica DM4000, Wetzlar, Germany) with the use of computer software (Image J, NIH, MD, USA; Adobe Photoshop, San Jose, CA, USA; and JV Analysis)

- Statistical Analysis

Statistical Analysis was conducted utilizing a general linear mixed model (GLMM) ANOVA (at a 95% confidence interval, 𝛼 = 0.05). The independent variables considered (individually or in combination) were the presence of grafting material and L-PRF. The dependent variable evaluated were BAFO, vertical/horizontal ridge dimensions, and area of coronal soft tissue infiltration.

## Results

**Figure 1:**
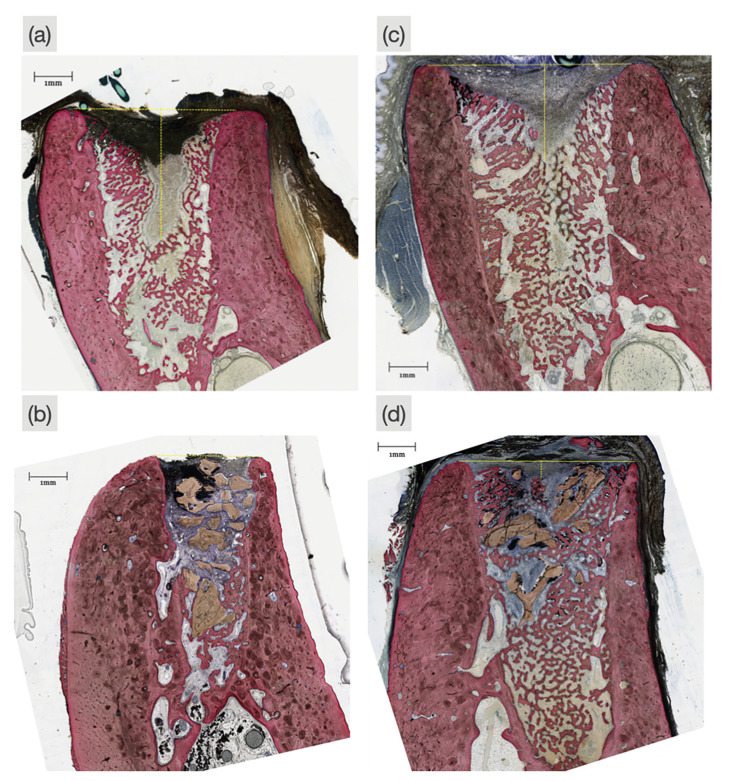
Representative histologic sections of post-extraction sites of (a) Empty socket, left for spontaneous healing as a negative control; (b) Bio-Oss®, where the socket was filled with Bio-Oss® grafting material; (c) L-PRF, where an L-PRF membrane was used to fill the extraction site; and (d) OSS w/L-PRF, where the socket was filled with a mixture of Bio-Oss® and L-PRF.

No operative or post-operative complications were observed during the study. Histological assessment reveals extraction sites with minimal trauma, intact buccal/lingual walls, and intramembranous-like healing in which bone regeneration occurred from the bone walls and migrated towards the center (Fig. 1).

Histological evaluation of the control group, revealed newly regenerated bone characterized as woven bone due to its irregular collagen fiber arrangement and abundant presence of osteoblasts releasing an osteoid matrix. In contrast, the L-PRF filled defects yielded a higher level of parallel alignment of collagen fibers, decreased number of osteoblasts, and presence of cutting cones produced by osteoclasts, indicating bone remodeling. Both the Bio-Oss® filled and the Bio-Oss® w/L-PRF filled defects displayed bone regeneration along the graft particle surface, but the later presented less soft tissue encapsulation, further bone regeneration along the graft surface, and mature bone morphology. Compared to the control group, the defects filled with Bio-Oss® or L-PRF alone had prevented vertical collapse of the alveolar ridge and halted the process of progressive coronal soft tissue infiltration.

**Figure 2:**
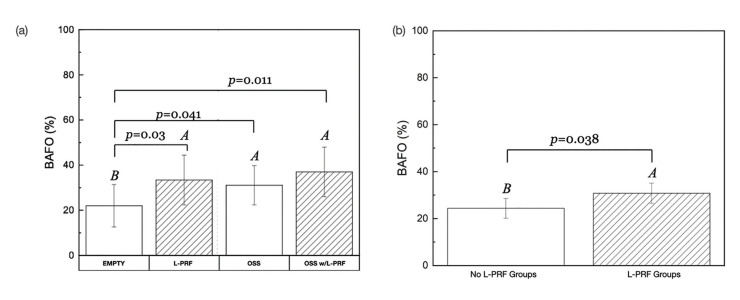
Statistical summary of (A) BAFO evaluating the effect of L-PRF and grafting material, and of (B) BAFO when collapsed across all groups.

BAFO quantitative evaluation as a function of grafting material showed that L-PRF filled defects depicted a significantly higher BAFO (*p* = 0.03) in comparison to the control group. A statistically significant difference was detected for negative controls compared to the defects treated with Bio-Oss® (*p* = .04) and Bio-Oss® with L-PRF (*p* = .01). No statistical difference was detected for the defects filled with Bio-Oss® w/L-PRF compared to Bio-Oss® (*p* = 0.13) and L-PRF (*p* = 0.26). L-PRF filled defects did not yield a significant difference (*p* = 0.33) in BAFO compared to those filled with Bio-Oss® (Fig. 2). Collapsed across all groups, defects filled with L-PRF exhibited statistically greater means of BAFO than those without L-PRF (*p* = 0.03) (Fig. 2).

**Figure 3:**
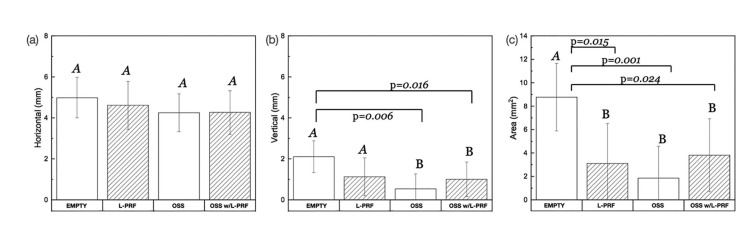
Statistical summary of (A) Horizontal ridge dimensions, (B) Vertical ridge reduction and (C) Area of coronal soft tissue infiltration of all evaluated groups.

**Figure 4:**
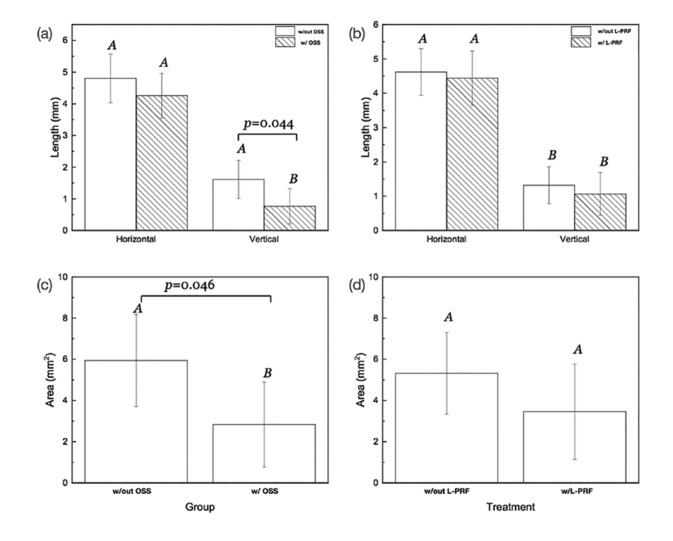
Statistical summary of (A-B) Vertical ridge reduction and Horizontal ridge dimensions and (C-D) Area of coronal soft tissue infiltration when collapsed across all groups.

Evaluation of vertical ridge dimensions as a function of grafting material and the presence of L-PRF depicted that Bio-Oss® with and without L-PRF significantly maintained vertical ridge dimensions compared to the control group (*p* = 0.02 and 0.01, respectively) (Fig. 3). Collapsed across all groups, Bio-Oss® filled defects better maintained the vertical ridge dimension and resulted in less area of coronal soft tissue infiltration compared to the control group (*p* = 0.04 and *p* = 0.04) (Fig. 4).

Furthermore, evaluation of the area of coronal soft tissue infiltration noted a significant difference for L-PRF, Bio-Oss® and Bio-Oss® w/L-PRF compared to the empty defect (*p* = 0.015, 0.024, and 0.001, respectively) (Fig. 3). However, we observed no difference in area of coronal soft tissue infiltration for the L-PRF filled defect (Fig. 4). Lastly, no significant differences in horizontal ridge dimensions were observed among the study groups (Fig. 3).

## Discussion

Several studies have reported positive results on the use of L-PRF associated with grafting material (17). However, limited literature regarding histological and histomorphometrical events is available on the use of L-PRF alone. When used individually for socket preservation, L-PRF has shown to yield greater amount of bone formation in comparison to PRP or PPP, potentially due to the dense fibrin network provided by L-PRF serving as a scaffold for cells and growth factors (13). Its dense fibrous network may play a role in space making for bone regeneration (18). In addition, the L-PRF low level of thrombin allows for optimum migration of endothelial cells and fibroblasts (8).

The histomorphometric results indicated that L-PRF, when used alone as a grafting material, yielded a significant increase in BAFO compared to the control group. These results can be associated to the fibrin network provided by L-PRF, allowing for regenerative sites for cellular migration and vascularization. Moreover, the platelet derived cytokines (i.e., PDGF, TGF-β, and IGF-1), that are continuously released from the fibrin matrix, have the potential to promote healing properties and regulation of inflammatory processes (19). Histological examination further depicted greater migration of osteoblasts and initiation of angiogenesis. Extraction sites augmented with L-PRF displayed bone qualities normally seen in lamellar bone such as the presence of parallel fiber arrangement, cutting cones, with increased remodeling activity. Such activity/results may be linked to the high concentration of growth factors present in platelet concentrates that lead to increased cell signaling, ultimately resulting in cell proliferation, chemotaxis, and migration, which are events necessary for bone remodeling. This phenomenon suggests that L-PRF is viable as a material for hastening bone healing and remodeling.

The combination of particulate bone grafts with L-PRF have been explored due to its possible synergistic effects in improving bone regeneration. In our study, the Bio-Oss® material did not exhibit significantly higher BAFO when combined with L-PRF. This observation agrees with literature and may be explained by the absence of progenitor cells in Bio-Oss® (6). On the other hand, the OSS w/L-PRF group demonstrated a higher maintenance of the vertical ridge height in our study. This may be attributed to the role of L-PRF as a membrane scaffold and a reservoir for growth factors. Direct apposition of newly mineralized bone to the Bio-Oss® material was seen at 6 weeks, which is a stage necessary for gradual tissue replacement with autogenous bone. Other studies have reported that combination of grafting material and L-PRF to hasten soft and hard tissue healing (6,20). No statistical difference was found for alveolar socket regeneration among defects filled with L-PRF versus those filled with Bio-Oss® with or without L-PRF. This finding suggests that L-PRF alone could promote alveolar socket regeneration to levels comparable to particulate xenografts associated or not with L-PRF membranes.

Previous studies have suggested that materials such as bone grafts and L-PRF may reduce both horizontal and vertical bone loss, which is crucial for alveolar ridge dimensional stability. A systematic review of hard and soft tissue dimensional changes pointed out that horizontal reduction was greater than the vertical reduction at 6 months following teeth extraction, with most rapid reductions occurring in the first 3-6 months (21). Particulate xenografts have a well stablished osteoconductive capabilities and slow resorption time (5,22). Likewise, in our study, the defects filled with Bio-Oss® with and without L-PRF presented with less vertical ridge collapse compared to the control group. L-PRF has a weaker mechanical stability and quicker resorption time in comparison to xenogeneic bone grafting materials. The L-PRF filled defects did not have a statistically significant difference in vertical ridge height compared to the control group, but we did observe a relatively lower mean vertical ridge reduction.

Bovine particulate graft acts as a space maintainer preventing migration of soft tissue into the socket (23). Moreover, L-PRF’s role in enhancing biological responses has been credited for preventing soft tissue encapsulation of particulate grafts and encouraging bone formation and remodeling processes. Our treatment groups presented significantly less area of coronal soft tissue infiltration compared to the control group.

Different centrifugation protocols have been described to obtain L-PRF. Studies have reported that different centrifuge hardware, including the use of *in vitro* diagnostic devices, affect the quality and quantity of the L-PRF produced as they yield different levels of vibrations despite utilizing identical speed and protocol (24). The centrifugation speed to obtain an ideal L-PRS is still an object of debate in the scientific community. On one hand, the slow-speed centrifugation is associated with increased release of growth factors and a more uniform cell distribution within the clots (25). On the other hand, lower relative centrifugal forces have also been associated with smaller clots with a weak fibrin matrix, which may compromise the L-PRF efficacy for bone regeneration (24). A recently published pre-clinical In-vivo study suggest that L-PRF clots obtained at higher relative centrifugal forces provide a stronger fibrin matrix that facilitate bone regeneration (26). Therefore, it is therefore imperative that similar protocols should be followed if the clinical applications of L-PRF are to be comparable.

The present study comes with limitations. Evaluation of the horizontal ridge dimensions resulted in no significant differences in our study. This can be expected as the buccal and lingual external cortical plates of the socket walls remain intact at 6 weeks, which was our study’s endpoint. Further studies at later time points will be necessary to fully evaluate the effects of socket preservation on horizontal ridge augmentation, as well as evaluate the effects of L-PRF at different stages of osteoblastic differentiation. Due to the inherent difference in degradation time and osteogenic capacities between grafting materials, different time points will be necessary to assess early and late healing processes. However, our study was designed to evaluate the effects of L-PRF in combination with a particulate xenograft material. The rationale was to systematically evaluate the effects of L-PRF membranes in alveolar ridge preservation with and without grafting material, while standardizing outside parameters such as the age, sex, weight, bone/tissue characteristics, physical activity, and hormonal status of the study subjects. Additional parameters, including design/duration of study, surgical protocols, and supervision of post-operative mobilization were also controlled. Due to the significant time, cost, and potential risks associated with clinical studies, pre-clinical *in vivo* studies are desirable to assess safety, efficacy, regenerative potential, and immune response prior to clinical applications. The study was designed with a split-mouth arrangement in bilateral model allowing for a direct comparison between groups nested within the same subject. The postulated hypothesis, that the combination of L-PRF with grafting material would result in higher bone regeneration was not accepted as no further bone healing was achieved by adding L-PRF to particulate xenograft at 6 weeks *in vivo*.

## Conclusions

No further bone healing is achieved by adding L-PRF to particulate xenograft at 6 weeks *in vivo*. L-PRF, when utilized alone, augments alveolar socket regeneration to levels comparable to particulate xenografts. This suggests that L-PRF is a viable material for socket preservation. Additionally, xenograft material better preserved the vertical ridge dimension, while L-PRF resulted in comparable less area of coronal soft tissue infiltration. Further investigation is warranted at different time points to investigate early and late bone healing stages.

## References

[1] Irinakis T (2006). Rationale for socket preservation after extraction of a single-rooted tooth when planning for future implant placement. J Can Dent Assoc.

[2] Pagni G, Pellegrini G, Giannobile WV, Rasperini G (2012). Postextraction alveolar ridge preservation: biological basis and treatments. Int J Dent.

[3] Wang W, Yeung KWK (2017). Bone grafts and biomaterials substitutes for bone defect repair: A review. Bioact Mater.

[4] Kumar P, Vinitha B, Fathima G (2013). Bone grafts in dentistry. J Pharm Bioallied Sci.

[5] Jensen SS, Aaboe M, Pinholt EM, Hjorting-Hansen E, Melsen F, Ruyter IE (1996). Tissue reaction and material characteristics of four bone substitutes. Int J Oral Maxillofac Implants.

[6] Inchingolo F, Tatullo M, Marrelli M, Inchingolo AM, Scacco S, Inchingolo AD (2010). Trial with Platelet-Rich Fibrin and Bio-Oss used as grafting materials in the treatment of the severe maxillar bone atrophy: clinical and radiological evaluations. Eur Rev Med Pharmacol Sci.

[7] de Oliveira P, Bonfante EA, Bergamo ETP, de Souza SLS, Riella L, Torroni A (2020). Obesity/Metabolic Syndrome and Diabetes Mellitus on Peri-implantitis. Trends Endocrinol Metab.

[8] Prakash S, Thakur A (2011). Platelet concentrates: past, present and future. J Maxillofac Oral Surg.

[9] Matras H (1985). Fibrin seal: the state of the art. J Oral Maxillofac Surg.

[10] Marx RE, Carlson ER, Eichstaedt RM, Schimmele SR, Strauss JE, Georgeff KR (1998). Platelet-rich plasma: Growth factor enhancement for bone grafts. Oral Surg Oral Med Oral Pathol Oral Radiol Endod.

[11] Canellas J, Medeiros PJD, Figueredo C, Fischer RG, Ritto FG (2019). Platelet-rich fibrin in oral surgical procedures: a systematic review and meta-analysis. Int J Oral Maxillofac Surg.

[12] Choukroun J, Diss A, Simonpieri A, Girard MO, Schoeffler C, Dohan SL (2006). Platelet-rich fibrin (PRF): a second-generation platelet concentrate. Part IV: clinical effects on tissue healing. Oral Surg Oral Med Oral Pathol Oral Radiol Endod.

[13] Dohan Ehrenfest DM, Andia I, Zumstein MA, Zhang CQ, Pinto NR, Bielecki T (2014). Classification of platelet concentrates (Platelet-Rich Plasma-PRP, Platelet-Rich Fibrin-PRF) for topical and infiltrative use in orthopedic and sports medicine: current consensus, clinical implications and perspectives. Muscles Ligaments Tendons J.

[14] Su CY, Kuo YP, Tseng YH, Su CH, Burnouf T (2009). In vitro release of growth factors from platelet-rich fibrin (PRF): a proposal to optimize the clinical applications of PRF. Oral Surg Oral Med Oral Pathol Oral Radiol Endod.

[15] Castro AB, Meschi N, Temmerman A, Pinto N, Lambrechts P, Teughels W (2017). Regenerative potential of leucocyte- and platelet-rich fibrin. Part A: intra-bony defects, furcation defects and periodontal plastic surgery. A systematic review and meta-analysis. J Clin Periodontol.

[16] Dohan DM, Choukroun J, Diss A, Dohan SL, Dohan AJ, Mouhyi J (2006). Platelet-rich fibrin (PRF): a second-generation platelet concentrate. Part I: technological concepts and evolution. Oral Surg Oral Med Oral Pathol Oral Radiol Endod.

[17] Choukroun J, Diss A, Simonpieri A, Girard MO, Schoeffler C, Dohan SL (2006). Platelet-rich fibrin (PRF): a second-generation platelet concentrate. Part V: histologic evaluations of PRF effects on bone allograft maturation in sinus lift. Oral Surg Oral Med Oral Pathol Oral Radiol Endod.

[18] Hatakeyama I, Marukawa E, Takahashi Y, Omura K (2014). Effects of platelet-poor plasma, platelet-rich plasma, and platelet-rich fibrin on healing of extraction sockets with buccal dehiscence in dogs. Tissue Eng Part A.

[19] Simonpieri A, Del Corso M, Sammartino G, Dohan Ehrenfest DM (2009). The relevance of Choukroun's platelet-rich fibrin and metronidazole during complex maxillary rehabilitations using bone allograft. Part II: implant surgery, prosthodontics, and survival. Implant Dent.

[20] Miron RJ, Fujioka-Kobayashi M, Moraschini V, Zhang Y, Gruber R, Wang HL (2021). Efficacy of platelet-rich fibrin on bone formation, part 1: Alveolar ridge preservation. Int J Oral Implantol (Berl).

[21] Tan WL, Wong TL, Wong MC, Lang NP (2012). A systematic review of post-extractional alveolar hard and soft tissue dimensional changes in humans. Clin Oral Implants Res.

[22] Liu J, Kerns DG (2014). Mechanisms of guided bone regeneration: a review. Open Dent J.

[23] Bunyaratavej P, Wang HL (2001). Collagen membranes: a review. J Periodontol.

[24] Dohan Ehrenfest DM, Pinto NR, Pereda A, Jimenez P, Corso MD, Kang BS (2018). The impact of the centrifuge characteristics and centrifugation protocols on the cells, growth factors, and fibrin architecture of a leukocyte- and platelet-rich fibrin (L-PRF) clot and membrane. Platelets.

[25] El Bagdadi K, Kubesch A, Yu X, Al-Maawi S, Orlowska A, Dias A (2019). Reduction of relative centrifugal forces increases growth factor release within solid platelet-rich-fibrin (PRF)-based matrices: a proof of concept of LSCC (low speed centrifugation concept). Eur J Trauma Emerg Surg.

[26] Tovar N, Benalcazar Jalkh EB, Ramalho IS, Rodriguez Colon R, Kim H, Bonfante EA (2021). Effects of relative centrifugation force on L-PRF: An in vivo submandibular boney defect regeneration study. J Biomed Mater Res B Appl Biomater.

